# The Effect of Ketamine on Electrophysiological Connectivity in Major Depressive Disorder

**DOI:** 10.3389/fpsyt.2020.00519

**Published:** 2020-06-10

**Authors:** Allison C. Nugent, Elizabeth D. Ballard, Jessica R. Gilbert, Prejaas K. Tewarie, Matthew J. Brookes, Carlos A. Zarate

**Affiliations:** ^1^MEG Core Facility, National Institute of Mental Health, National Institutes of Health, Bethesda, MD, United States; ^2^Experimental Therapeutics and Pathophysiology Branch, National Institute of Mental Health, National Institutes of Health, Bethesda, MD, United States; ^3^Sir Peter Mansfield Imaging Centre, School of Physics and Astronomy, University of Nottingham, Nottingham, United Kingdom

**Keywords:** magnetoencephalography, resting state, network, connectivity, depression

## Abstract

Major depressive disorder (MDD) is highly prevalent and frequently disabling. Only about 30% of patients respond to a first-line antidepressant treatment, and around 30% of patients are classified as “treatment-resistant” after failing to respond to multiple adequate trials. While most antidepressants target monoaminergic targets, ketamine is an N-methyl-D-aspartate (NMDA) antagonist that has shown rapid antidepressant effects when delivered intravenously or intranasally. While there is evidence that ketamine exerts its effects *via* enhanced α-amino-3-hydroxy-5-methyl-4-isoxazolepropionic acid (AMPA) throughput, its mechanism for relieving depressive symptoms is largely unknown. This study acquired resting-state magnetoencephalography (MEG) recordings after both ketamine and placebo infusions and investigated functional connectivity using a multilayer amplitude-amplitude correlation technique spanning the canonical frequency bands. Twenty-four healthy volunteers (HVs) and 27 unmedicated participants with MDD took part in a double-blind, placebo-controlled, crossover trial of 0.5 mg/kg IV ketamine. Order of infusion was randomized, and participants crossed over to receive the second infusion after two weeks. The results indicated widespread ketamine-induced reductions in connectivity in the alpha and beta bands that did not correlate with magnitude of antidepressant response. In contrast, the magnitude of ketamine's antidepressant effects in MDD participants was associated with cross-frequency connectivity for delta-alpha and delta-gamma bands, with HVs and ketamine non-responders showing connectivity decreases post-ketamine and ketamine responders demonstrating small increases in connectivity. These results may indicate functional subtypes of MDD and also suggest that neural responses to ketamine are fundamentally different between responders and non-responders.

## Introduction

Major depressive disorder (MDD) affects up to 20% of people at some point across their lifespan and is associated with significantly increased morbidity and mortality. Yet MDD remains difficult to treat; most conventional antidepressant medications take weeks to achieve their maximum effects, and only 30% of those with MDD respond to a first-line antidepressant (1). In contrast, the N-methyl-D-aspartate (NMDA) antagonist ketamine, a racemic mixture of ketamine's *R*- and *S*-isomers, has rapid antidepressant effects when delivered *via* either infusion or nasal spray. Notably, the discovery of ketamine's antidepressant properties and the subsequent FDA approval of esketamine (the *S*-isomer of ketamine) in March 2019 represents the first novel target for MDD since the development of selective serotonin reuptake inhibitors (SSRIs). Nevertheless, relatively little is known about how ketamine exerts its antidepressant effects. Evidence suggests that ketamine binds to receptors on both excitatory glutamatergic pyramidal neurons as well as inhibitory gamma aminobutyric acid (GABA)-ergic interneurons. This leads to disinhibition of the pyramidal neurons, resulting in a glutamate surge in the synapse and enhanced α-amino-3-hydroxy-5-methyl-4-isoxazolepropionic acid (AMPA) throughput, leading to downstream changes that enhance synaptic plasticity (2).

While numerous studies have investigated ketamine's effects on brain connectivity while participants performed a variety of cognitive tasks, examining resting-state connectivity may reveal more pervasive abnormalities not limited to any one cognitive domain. Most of the extant literature examining functional connectivity changes induced by ketamine were performed using functional magnetic resonance imaging (fMRI). However, a significant limitation of the fMRI literature is the fact that ketamine has prominent cardiovascular effects that can also alter the blood oxygen level dependent (BOLD) fMRI signal. Thus, ketamine's inherent neural effects cannot be completely disambiguated from its cardiovascular effects using a hemodynamic-based imaging technique. As a result, fMRI analyses are extremely sensitive to the type of pre-processing used to remove physiological effects, and differences in pre-processing pipelines may yield different results with regard to connectivity changes (up *versus* down) post-ketamine (3). The significant heterogeneity in the literature is therefore not surprising. With regard to healthy volunteers (HVs), some studies that used resting-state fMRI to examine ketamine's acute effects found primarily increased connectivity (4–7), others reported mainly reductions (8–10), and some reported patterns of increases and decreases in connectivity depending on the brain region examined (11–14). Studies of functional connectivity between one hour and 24 h post-infusion have produced similarly heterogeneous results (15–20). As regards individuals with MDD, extant studies that measured resting-state connectivity have observed increased default mode network (DMN) connectivity (5), decreased dorsal anterior cingulate cortex (dACC) connectivity (21), and a positive correlation between increased subgenual ACC (sgACC) connectivity post-ketamine and symptom reduction (16). While investigations of global brain connectivity (GBC) have primarily found post-ketamine increases in connectivity, particularly in the prefrontal cortex (PFC) (20, 22, 23), these studies may be especially susceptible to pre-processing strategy (24).

Despite the inherent issues associated with hemodynamic-based functional imaging methods, relatively few electrophysiological investigations of ketamine's effects on connectivity have been conducted. One study reported decreased alpha-band phase locking during ketamine-induced anesthesia in participants undergoing elective surgery (25). Another study found that *S*-ketamine increased broad-band transfer entropy, a measure of information transfer, in regions showing increased gamma or decreased beta power in HVs during ketamine infusion (26). A dynamic causal modeling (DCM) study, which modeled observed resting-state neuromagnetic connectivity using a biophysical model of neuronal responses incorporating AMPA and NMDA connectivity parameters, found an acute decrease in AMPA and NMDA-mediated frontal to parietal connectivity in HVs (27); the same study observed reductions in alpha- and beta-band amplitude envelope connectivity in visual parietal and motor networks. In addition, another electroencephalography (EEG) study demonstrated significant and widespread decreases in amplitude envelope connectivity in the alpha-band, particularly for nodes within the occipital lobe, as well as between the occipital lobe and frontal, parietal, and temporal nodes (3). Decreases in low beta-band connectivity have also been observed in healthy male participants, primarily in motor areas (3). These findings are consistent with results of a MEG study conducted in MDD participants that observed decreased beta-band amplitude envelope connectivity between the sgACC and a bilateral precentral network, as well as between bilateral amygdala and insulo-temporal nodes several hours post-ketamine infusion (28). Thus, in contrast to findings from the fMRI literature, ketamine-induced changes in connectivity measured using electrophysiological techniques are far more convergent and point to decreases in connectivity, particularly in the alpha- and beta- bands.

The present study acquired MEG recordings in both HVs and individuals with MDD 6 to 9 h post-ketamine and placebo infusions in a double-blind, placebo-controlled, crossover trial. The study sought to characterize changes in connectivity induced by ketamine infusion using a multi-layer network approach (29) that examined band-limited amplitude envelope correlations within and between the canonical frequency bands delta, theta, alpha, beta, and gamma. Based on prior literature, we expected to see reductions in alpha- and beta-band connectivity following ketamine *versus* placebo infusions in the three core networks central to the pathophysiology of MDD: the DMN, the central executive network (CEN), and the salience network (SN). The study also examined how changes in connectivity are related to antidepressant response to ketamine in participants with MDD. This study is unique in its inclusion of both unmedicated MDD participants and HVs, its use of placebo-controlled infusions, and its use of MEG to enable spatial localization of connectivity changes.

## Materials and Methods

### Participants

Twenty-four HVs and 30 participants with MDD took part in a double-blind, placebo-controlled, crossover trial of 0.5 mg/kg IV ketamine (NCT00088699) and were included in this study. Order of infusion was randomized, and participants crossed over to receive the second infusion after 2 weeks. Clinical data on the full sample have been reported previously, along with findings involving oscillatory power changes post-ketamine (30). A previous study assessing baseline differences was also performed using comparable methods to those employed herein (manuscript under revision).

A diagnosis of MDD was established using the Structured Clinical Interview for DSM-IV-TR (SCID) and an unstructured interview with a study psychiatrist. For inclusion in the study, MDD participants had to have a Montgomery-Åsberg Depression Rating Scale [MADRS, (31)] score of at least 20 at baseline, and they did not cross over to the second infusion unless MADRS score was above 20. All MDD participants had not responded to at least one adequate antidepressant trial during their current episode, as assessed using the Antidepressant Treatment History Form (32), and the current episode had to have lasted 4 weeks. HVs had no personal or first-degree family history of mood disorders. All participants were free of medications thought to impact central nervous system function, including antidepressants, for at least 2 weeks (5 weeks for fluoxetine, 3 weeks for aripiprazole) and were medically healthy. The study was approved by the NIH combined CNS IRB, and all participants gave written informed consent.

### Clinical Intervention and Data Acquisition

Participants were randomized to receive an infusion of either saline or 0.5 mg/kg IV ketamine over 40 min under double-blind conditions. Two weeks after the first infusion, participants crossed over to receive the second infusion. Depressive symptoms were assessed using the MADRS at 60 min prior to each infusion (t = −60); at 40, 80, 120, and 230 min post-infusion on the day of the infusion; and on Days 1, 2, 3, 7, 10, and 11 post-infusion.

MEG recordings were acquired on a 275 channel CTF system (Coquitlam, BC) using synthetic third gradient correction to remove environmental noise approximately 6 to 9 h post-infusion. One or two recordings were acquired during each session at 1,200 Hz with a 300 Hz bandwidth. Participants were instructed to relax with their eyes closed and minimize movement. T1-weighted MRI scans were acquired on a 3T GE scanner for co-registration.

## MRI Data Pre-Processing

As reported previously (manuscript under revision), a series of 34 regions of interest (ROIs) was defined on a Talairach template. These ROIs encompassed major nodes in the three networks of the triple network model (33), namely the DMN, SN, and CEN. Other ROIs included visual and motor network nodes, subcortical regions (hippocampus, amygdala, and thalamus), and depression-specific regions such as the sgACC, pregenual ACC (pgACC), and orbital cortex areas. The central coordinate of each node was transformed to the participants' native MRIs, and 7.5 mm-radius ROIs were constructed. MEG recordings were localized to the MRI scan using three fiducial coils placed at standard locations on the participants' heads.

### MEG Data Pre-Processing

MEG data were pre-processed as previously reported (manuscript under revision). Briefly, datasets were first processed using an independent components analysis (ICA) to remove any artifactual components. Datasets with more than 10 artifactual components were removed from further analysis. A covariance matrix was calculated across the recording in a 2–100 Hz bandwidth, and synthetic aperture magnetometry [SAM, (34)] beamformer weights were calculated. A Hilbert envelope time series was calculated in the five canonical frequency bands: delta (δ, 2–4 Hz), theta (θ, 4–8 Hz), alpha (α, 8–14 Hz), beta (β, 14–30 Hz), and gamma (γ, 30–55 Hz). An “artifact gamma” time series in the 200–235 Hz band was also calculated to correct for contamination with muscular artifacts. The mean time series was extracted for each of the 34 ROIs, which were then symmetrically orthogonalized to minimize leakage effects (35). The approach to connectivity follows the multi-level strategy employed by Brookes and colleagues (29). Correlation between nodes was calculated for Hilbert envelope time series within each frequency band (i.e. alpha-to-alpha amplitude connectivity, denoted α−α) as well as between frequency bands (i.e. alpha-amplitude-to-beta-amplitude connectivity, denoted α−β), forming matrices. While the lower frequency in each pair is presented first by convention, connectivity was non-directional. These “tiles” were arrayed in a “super-adjacency” matrix. For each tile, the mean over the entire tile was calculated. In order to remove participants with spurious connectivity due to correlated noise, datasets were removed if the mean connectivity in any of the within-frequency tiles exceeded the overall grand mean by four standard deviations. When reporting the findings, connectivity tiles are referred to using Greek lettering; however, when referring to the results of other investigators who may have used significantly different methodologies, frequency bands will continue to be spelled out.

### Statistical Data Analysis

Because clinical response to ketamine has been reported previously for this sample (30), only mean responses are given for the subgroup of participants included in this analysis.

The initial statistical analysis on the connectivity data was performed on the means of each tile. Linear mixed models were performed with SPSS software, using an unstructured covariance matrix. Drug session (Drug), diagnosis (DX), and the Drug*DX interaction were effects of interest. Because we attempted to acquire two usable resting state recordings for each participant, an additional factor was added to encode whether the recording occurred at the beginning and end of a session (before or after additional cognitive tests). Age and gender were additional effects of no interest included in the model. Tiles that showed Drug, DX, or Drug*DX effects at p < 0.05/15 (to correct for the 15 unique tiles in the super-adjacency matrix) are reported. This analysis was repeated by dividing the MDD group into responders and non-responders, retaining the HV group in the analysis to determine if responders and non-responders differed from HVs.

Additional exploratory analyses were carried out to determine whether diagnostic groups differed in the relationship between change in connectivity between ketamine and placebo sessions and change in MADRS score post-ketamine. Because this analysis incorporated mood rating changes in both MDD participants and HVs, absolute change in MADRS score was used. The 40-min post-infusion timepoint was chosen as the point at which change in MADRS score in response to ketamine was largest in both diagnostic categories. Because the outcome measure here was the difference in connectivity between ketamine and placebo sessions, connectivity was averaged over multiple recordings in the same session. Thus, the effects of interest in the model were DX, MADRS change, and DX*MADRS. Age and gender were included as main effects only. Finally, a similar analysis was performed in the MDD sample only, to assess connectivity correlates of the antidepressant effect. For this analysis, percent change in MADRS score at Day 1 was chosen because it is the most commonly used metric for assessing antidepressant response to ketamine in MDD. For these exploratory analyses, tiles that showed a significant relationship with MADRS response at p < 0.05 are reported.

These same tests were repeated over the entire super-adjacency matrices using linear mixed models as implemented in AFNI's 3dLME program. The threshold was set at a false discovery rate (FDR) corrected p_FDR_ < 0.05. For the exploratory analysis assessing relationship to antidepressant response, if no connections survived FDR correction across the entire super-adjacency matrix, tiles that showed effects in the mean connectivity analysis were examined individually with FDR correction applied only over the tile. To aid in visualization of the cross-frequency tiles, models with significant findings were also repeated using a reduced version of the super-adjacency matrix where ROIs in each network-based group were averaged, resulting in connectivity graphs between DMN, CEN, SN, visual, motor, subcortical, and depression-related nodes.

## Results

### Participants

A total of 180 recordings, drawn from 30 MDD participants and 24 HVs, were initially included in the analysis. Some participants only had usable ketamine recordings and others only had usable placebo recordings. Five recordings were dropped from the analysis because there were more than 10 artifactual components present in the ICA decomposition. Four ketamine recordings were dropped due to excessive movement, and 17 recordings were dropped due to outlying mean within-frequency connectivity (as detailed in the *Methods*). After calculating differences in connectivity between placebo and ketamine sessions, one MDD participant demonstrated an extreme difference between ketamine and placebo sessions (greater than eight standard deviations above the mean of all other participants) and was therefore dropped from all analyses (including one ketamine recording and two placebo recordings). Thus, the final dataset comprised 151 recordings from 27 MDD participants and 24 HVs. Twenty-three of the MDD participants and 20 HVs had usable ketamine recordings, and 22 of the MDD participants and 23 of the HVs had usable placebo recordings. Demographic information is given in **Table 1**.

**Table 1:** Demographic information for participants included in the analysis.

	N	Age (SD)	% Female	Baseline MADRS (SD)
HV	24	34.4 (10.7)	62.5	
MDD	27	36.8 (9.8)	63.0	32.5 (4.8)

HV, healthy volunteer; MDD, major depressive disorder; MADRS, Montgomery-Asberg Depression Rating Scale.

### Clinical Response

Consistent with our prior findings (30), MDD participants showed a robust decrease in MADRS score from baseline to 40 min (reduction of 11.7 ± 7.6 points), while HVs exhibited an increase in MADRS score from baseline at 40 min post-infusion (7.70 ± 5.31 points). A more commonly used metric for measuring antidepressant response is percent change in MADRS score, which showed a decrease in depressive symptoms in MDD participants at Day 1 (30.0 ± 33.6%).

### Mean Connectivity Within Tiles

**Figure 1:**
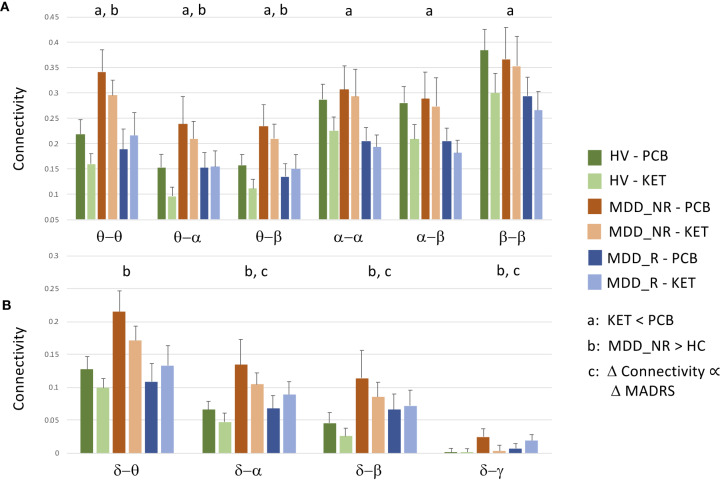
Raw data and standard errors for mean connectivity post-placebo (PCB) and post-ketamine for healthy volunteers (HVs), participants with major depressive disorder (MDD) who experienced an at least 30% reduction in depressive symptoms (MDD_R), and participants with MDD who experienced a less than 30% reduction in symptoms (MDD_NR). **(A)** Mean connectivity over tiles that showed significantly reduced connectivity post-ketamine compared to post-placebo. **(B)** Mean connectivity over tiles that showed no overall drug effect, but showed a diagnosis by change in Montgomery-Asberg Depression Rating Scale (MADRS) score interaction at t = 40 min post-infusion, a significant difference between HVs and MDD_NR, or a significant relationship between percent change in MADRS score at Day 1 in MDD participants alone.

Full results from the mixed models appear in **Table 2**, and mean values for selected tiles are plotted in **Figure 1**. Significant main effects of drug were observed for α−α connectivity (F(1,34) = 9.845, p = 0.003), α−β connectivity (F(1,40) = 17.715, p < 0.001), and β−β connectivity (F(1,34) = 15.786), p < 0.001). In all three cases, mean connectivity was reduced following ketamine infusion compared to placebo infusion. Although only ketamine *versus* placebo differences in connectivity at p < .05/15 =.0033 are reported, tiles whose mean values differed at p < 0.05 are also highlighted in **Table 2**.

**Table 2:** Mixed model results are shown for the primary analysis of mean connectivity over each within- or between-frequency tile.

	Drug	DX	DX*Drug
δ−δ	F(1,40)=0.374 p=0.544	F(1,43)=1.552 p=0.22	F(1,40)=0.013 p=0.91
δ−θ	F(1,41)=0.835 p=0.366	F(1,45)=3.875 p=0.055	F(1,42)=0.208 p=0.651
δ−α	F(1,44)=1.366 p=0.249	F(1,47)=4.817 p=0.033	F(1,41)=0.201 p=0.657
δ−β	F(1,42)=2.369 p=0.131	F(1,47)=5.689 p=0.021	F(1,41)=0.079 p=0.78
δ−γ	F(1,37)=2.823 p=0.101	F(1,43)=2.328 p=0.134	F(1,37)=0.205 p=0.654
θ−θ	F(1,37)=1.856 p=0.181	F(1,42)=3.699 p=0.061	F(1,37)=1.454 p=0.236
θ−α	F(1,40)=4.963 p=0.032	F(1,42)=2.691 p=0.108	F(1,40)=1.92 p=0.174
θ−β	F(1,39)=6.988 p=0.012	F(1,43)=3.952 p=0.053	F(1,40)=1.466 p=0.233
θ−γ	F(1,34)=0.786 p=0.381	F(1,43)=2.523 p=0.119	F(1,34)=0.312 p=0.58
α−α	F(1,34)=9.845 p=0.003	F(1,36)=0.01 p=0.919	F(1,29)=0.382 p=0.542
α−β	F(1,40)=17.715 p < 0.001	F(1,37)=0.048 p=0.828	F(1,29)=0.008 p=0.931
α−γ	F(1,34)=5.599 p=0.024	F(1,37)=0.939 p=0.339	F(1,34)=0.392 p=0.535
β−β	F(1,34)=15.786 p < 0.001	F(1,41)=0.032 p=0.859	F(1,32)=0.397 p=0.533
β−γ	F(1,28)=3.355 p=0.078	F(1,37)=1.901 p=0.176	F(1,30)=1.313 p=0.261
γ−γ	F(1,32)=0.015 p=0.903	F(1,43)=1.514 p=0.225	F(1,33)=0.175 p=0.679

Diagnosis (DX); major depressive disorder (MDD) and drug session (ketamine vs. placebo) were included as factors. Results significant at p < 0.05/15 =.0033 are highlighted in dark gray, while results for p < 0.05 are highlighted in light gray.

The previous analysis was repeated by dividing the MDD group into responders and non-responders (see **Table 3**). To obtain approximately equal numbers in each group, participants experiencing a greater than 30% reduction in depressive symptoms at Day 1 were classified as responders (N = 14), and those experiencing a 30% reduction or less were classified as non-responders (N = 12); note that one MDD participant only received the placebo infusion. While a 50% reduction is typically chosen to demonstrate efficacy and designate “responders,” the more lenient threshold of 30% was chosen because it signaled a clinically significant change in MADRS score and also helped achieve more balanced groups. This new diagnostic grouping variable is hereafter referred to as DX-Resp. Consistent with findings from the above analysis not stratified by clinical response, significant session effects were observed for α−β (F(1,40) = 16.862, p < 0.001) and β−β connectivity (F(1,34) = 12.758, p = 0.001). Although no significant DX-Resp*Drug interactions were observed (see **Table 3**, Column 4), there was a significant DX-Resp effect for θ−θ mediated connectivity (F(2,37) = 6.613, p = 0003; **Table 3**, Column 3). In *post-hoc* tests, θ−θ connectivity was significantly greater for MDD non-responders compared to both HVs (t(37) = 3.47, p = 0.001) and MDD responders (t(37) = 2.97, p = 0.005).

**Table 3:** Mixed model results are shown for the primary analysis of mean connectivity over each within- or between-frequency tile, where major depressive disorder (MDD) participants are stratified based upon a greater or lower than 30% reduction in depressive symptoms post-ketamine.

	Drug	DX-Responder	DX-Responder*Drug
δ−δ	F(1,40)=0.583 p=0.449	F(2,42)=4.335 p=0.019	F(2,40)=0.564 p=0.573
δ−θ	F(1,42)=0.873 p=0.356	F(2,43)=5.893 p=0.005	F(2,41)=1.763 p=0.184
δ−α	F(1,43)=1.374 p=0.248	F(2,47)=4.224 p=0.021	F(2,40)=0.789 p=0.461
δ−β	F(1,40)=2.072 p=0.158	F(2,46)=3.879 p=0.028	F(2,40)=0.555 p=0.579
δ−γ	F(1,37)=4.201 p=0.047	F(2,42)=1.248 p=0.298	F(2,36)=1.749 p=0.188
θ−θ	F(1,39)=1.193 p=0.281	F(2,37)=6.613 p=0.003	F(2,37)=1.920 p=0.161
θ−α	F(1,40)=3.886 p=0.056	F(2,43)=5.048 p=0.011	F(2,40)=1.609 p=0.213
θ−β	F(1,38)=5.706 p=0.022	F(2,44)=4.831 p=0.013	F(2,38)=1.839 p=0.173
θ−γ	F(1,35)=1.135 p=0.294	F(2,43)=2.188 p=0.125	F(2,34)=2.319 p=0.114
α−α	F(1,34)=8.402 p=0.007	F(2,35)=2.663 p=0.084	F(2,29)=0.265 p=0.769
α−β	F(1,40)=16.862 p < 0.001	F(2,37)=1.873 p=0.168	F(2,28)=0.087 p=0.917
α−γ	F(1,34)=4.581 p=0.04	F(2,37)=1.273 p=0.292	F(2,34)=0.288 p=0.752
β−β	F(1,34)=12.758 p=0.001	F(2,41)=1.32 p=0.278	F(2,31)=0.152 p=0.859
β−γ	F(1,29)=2.089 p=0.159	F(2,37)=1.843 p=0.173	F(2,30)=0.576 p=0.568
γ−γ	F(1,31)=0.0005 p=0.982	F(2,42)=0.611 p=0.547	F(2,32)=0.145 p=0.866

Results significant at p < 0.05/15 =.0033 are highlighted in dark gray, while results for p < 0.05 are highlighted in light gray.

DX, diagnosis.

In the exploratory analysis examining differences in connectivity between placebo and ketamine sessions with change in MADRS score at 40 minutes post-ketamine infusion, a DX*MADRS interaction was only found for δ−α (F(1,31) = 6.742, p = 0.014) and δ−β (F(1,31) = 6.621, p = 0.015). The δ−β connectivity model also showed a main effect of MADRS score (F(1,31) = 4.257, p = 0.048). Because no results would have survived our threshold for multiple comparisons, these analyses are not presented in table form. Surprisingly, for both δ−α and δ−β connectivity, MDD participants who showed an increase in connectivity post-ketamine compared to post-placebo tended to show the most robust responses, although MDD participants had nominally increased connectivity compared to HVs post-placebo. In addition, HVs who showed an increase in connectivity post-ketamine compared to post-placebo tended to show the greatest increase in depressive symptoms. In the MDD group alone, there were no significant relationships in any tiles between mean connectivity post-ketamine compared to post-placebo and percent change in MADRS score at Day 1, although a trend was observed in both δ−α (F(1,14) = 4.064), p = 0.063) and δ−β (F(1,14) = 3.21, p = 0.095) in the same direction as the 40-min analysis.

In order to disambiguate the relationship between ketamine and placebo connectivity values, as well as differences between depressed ketamine responders, depressed ketamine non-responders, and HVs, raw mean values were plotted for the tiles that showed the significant findings reported in this section, as well as for the tiles that showed significant individual connections in the next section. Thus, raw data are plotted for θ−θ, θ−α, θ−β, α−α, α−β, β−β in **Figure 1A**, and δ−θ, δ−α, δ−β, and δ−γ are plotted in **Figure 1B**.

### Super-Adjacency Matrices

**Figure 2:**
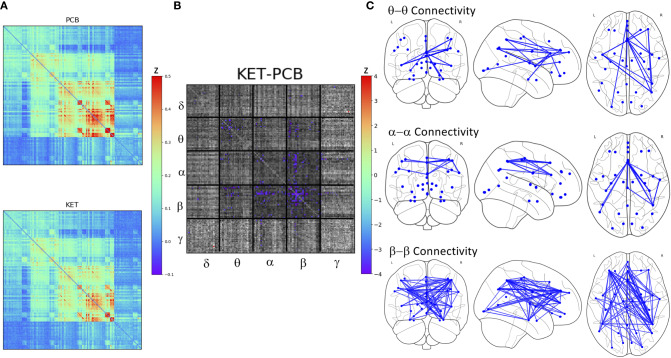
**(A)** Full super-adjacency matrices illustrating connectivity post-placebo and post-ketamine for all participants combined. **(B)** Full super-adjacency matrix for a *post-hoc t*-tests comparing ketamine to placebo connectivity, with connections showing significant differences highlighted in color. **(C)** Connections showing significant reductions in functional connectivity following ketamine compared to placebo (PCB) within the θ α and β bands.

**Figure 3:**
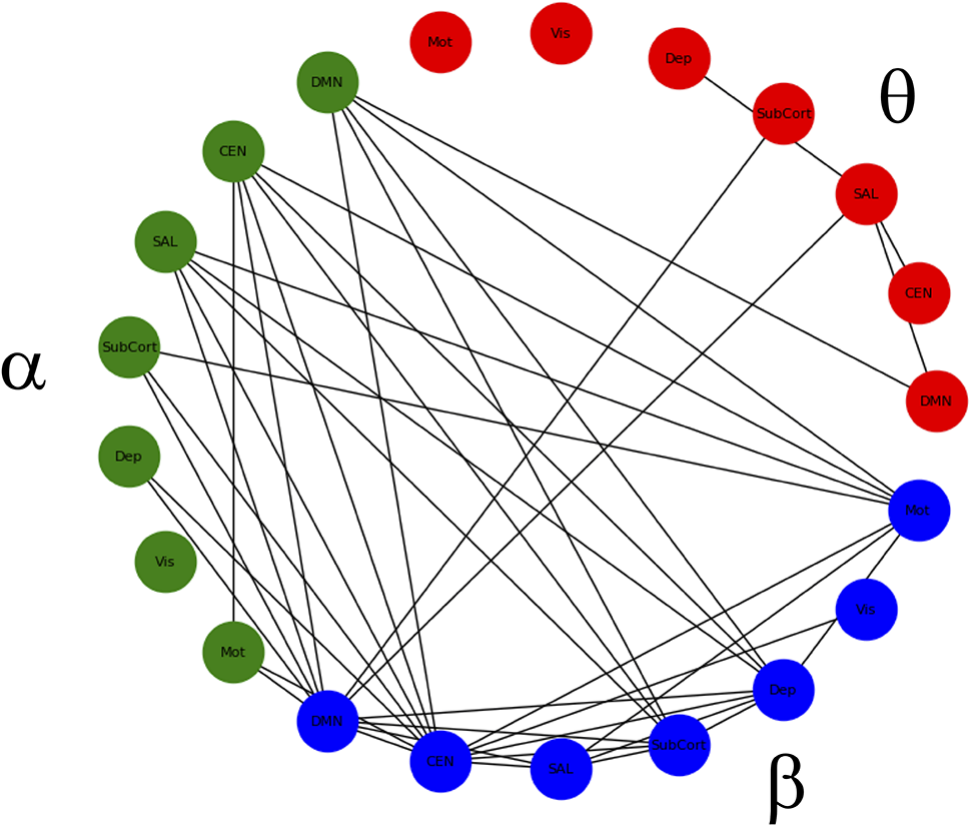
Significant within- and between-frequency connections between networks that were significantly reduced following ketamine infusion compared to placebo infusion.

There was no main effect of DX or DX*Drug interaction. There was a significant main effect of Drug. The result of a *post-hoc t*-test comparing ketamine to placebo is shown in **Figure 2**. The panel on the far right shows network maps for the within-frequency connections (θ−θ, α−α, and β−β). For θ−θ, the most prominently affected node was the dorsal cingulate. For α−α connections, the dorsomedial PFC (DMPFC) and right superior parietal cortex were most affected; both areas are in the CEN. For β−β connections, the left dorsolateral PFC (DLPFC), right superior parietal cortex, dorsal cingulate, right hippocampus, and pgACC were most reduced by ketamine. In order to visualize the cross-frequency interactions, the mixed-model analysis was repeated using the regions averaged over network group. The 50 connections showing the greatest magnitude Z-value reductions post-ketamine compared to post-placebo are plotted in **Figure 3**.

**Figure 4:**
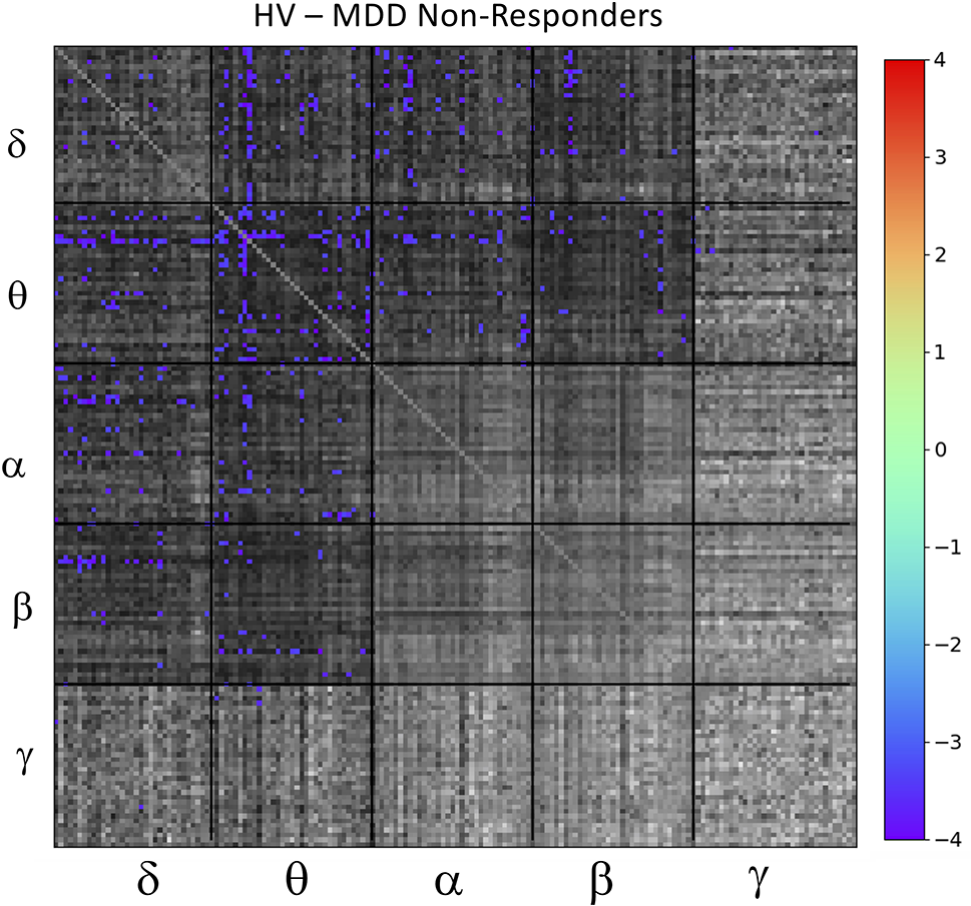
Full super-adjacency matrix illustrating significant differences in functional connectivity between healthy volunteers (HVs) and participants with major depressive disorder (MDD) who did not experience an antidepressant response to ketamine.

Based on results from the mean tile values investigating the main effect of DX-Resp, the super-adjacency analysis concentrated on the *post-hoc* contrast between HVs and ketamine non-responders. Due to the large number of connections at q < 0.05, and due to the small sample size, an additional uncorrected threshold of p < 0.001 was applied, corresponding to q < 0.0268. The ketamine non-responders differed most notably from the HVs in regions of the DMN and CEN, particularly for θ− θ and δ−θ connectivity (the full super-adjacency matrix appears in **Figure 4**, and individual θ−θ and δ−θ tiles are shown in **Supplementary Figure S1**). Given the small sample size, this analysis should be considered preliminary.

### Exploratory Analysis: Relationship Between Mood and Connectivity Changes

**Figure 5:**
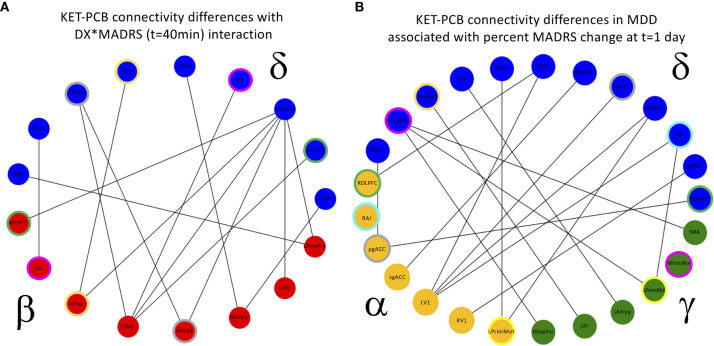
**(A)** Cross-frequency connections that showed a significant interaction between diagnosis (DX) and change in Montgomery-Asberg Depression Rating Scale (MADRS) score (t = 40 min). The interaction was such that the healthy volunteers (HVs) exhibiting the greatest increase in depressive symptoms showed increased functional connectivity, and the major depressive disorder (MDD) participants exhibiting the greatest decrease in depressive symptoms also showed increased functional connectivity. Colored rings indicate regions that appear in more than one frequency band. **(B)** Cross-frequency connections that showed a significant relationship with change in MADRS scores 1 day post-infusion in MDD participants alone. Again, participants exhibiting the greatest decrease in depressive symptoms also showed increased functional connectivity, and colored rings indicate regions that appear in more than one frequency band. PCB, placebo.

In the full super-adjacency matrix analysis modeling the difference in connectivity between ketamine and placebo sessions in both diagnostic groups with the addition of absolute change in MADRS score at 40 min, no connections survived FDR correction for multiple comparisons. Based on the results for the mean value of tiles, however, the δ−α and δ−δ tiles were examined more closely. Applying an FDR correction (q < 0.05) over all connections represented in each tile using a Benjamini-Hochberg procedure resulted in δ−β connections where change in connection strength was differentially correlated with 40-min change in MADRS score between HVs and MDD participants. Significant connections are illustrated in **Figure 5**.

Finally, the analysis was repeated in the MDD group alone, using percent change in MADRS score at Day 1 as the covariate of interest. In the analysis of the full super-adjacency matrix, connections survived FDR correction primarily in the δ−α and δ−γ tiles (the full super-adjacency matrix is shown in **Supplementary Figure S2**, and δ−α and δ−γ tiles are shown in **Figure 5**). These connections involved the interaction of motor and visual areas with higher cognitive areas, including the pgACC, right amygdala, right insula, DMPFC, and right DLPFC. Again, MDD participants who showed the most robust antidepressant response tended to show slight increases in connectivity, while those with a poor antidepressant response tended to show decreases. Mean δ−α and δ−γ connectivity was nominally increased in MDD participants compared to HVs, but this abnormality was far more prominent in the MDD non-responder group. Thus, while the MDD non-responders did indeed exhibit the greatest differences between ketamine and placebo recordings, they also demonstrated the greatest differences compared to HVs post-placebo (see **Figure 1B**).

## Discussion

Consistent with prior findings in the literature (3, 27, 28), results from the present study found that a single ketamine infusion produced robust and widespread reductions in β−β connectivity, irrespective of diagnosis. The present findings extend those prior results by demonstrating reductions in α−α and α−β cross-frequency connectivity, as well as θ−θ and θ−β cross-frequency connectivity. For θ−θ connections, the dorsal cingulate showed the most connections with reduced connectivity. In the α−α band, nodes involved in the CEN were particularly affected. For β−β connectivity, executive network regions as well as the dorsal cingulate, hippocampus, and pgACC showed the strongest reductions. For cross-frequency interactions, θ−β and α−β, the three core networks (CEN, DMN, and SN) figured prominently among the aberrant connections.

These results should be considered within the context of known mechanisms of cortical oscillation. Delta oscillations appear to underlie fundamental processes such as motivation and reward as well as homeostatic processes (36). Widespread decreases in intrinsic delta oscillations have been reported following ketamine infusion (27), particularly in the DMN (37). Theta oscillators are best studied in the hippocampus (38); however, theta rhythms can be generated in isolated sections of layer V pyramidal cells and depend on the activation of NMDA receptors (39). Ketamine's effects on theta may be regionally independent; for instance, one MEG study demonstrated increased theta anteriorly but decreased theta in posterior regions (27), and some studies have primarily reported reductions, particularly in the DMN and CEN (37). Despite its prominence in the human brain power spectrum, the alpha rhythm is also not fully understood, although current theories suggest that alpha oscillations provide functional inhibition (40), blocking pathways that are not relevant to the current task (41). As with theta, alpha oscillations require NMDA in the synapse (40). Ketamine has been shown to produce widespread decreases in both alpha power and connectivity (25, 27) or alpha power alone across all three core networks (DMN, CEN, SN) (37). Studies generally report that beta power is significantly reduced by ketamine infusion (26, 27), as is beta connectivity (27, 28). One study, however, reported that power changes were more regionally specific and might preferentially involve the SN (37), although this did not emerge in the current study as a particularly prominent site of ketamine's effects on beta-band connectivity. It is not at all clear, however, how these observed changes in amplitude relate to connectivity differences.

It is notable that the bands in which ketamine-induced reductions in functional connectivity were observed did not exhibit correlations between connectivity and clinical response. In an overlapping cohort at baseline, MDD participants exhibited nominally lower α−α, α−β, and β−β connectivity compared to HVs, which would imply that additional reductions induced by ketamine resulted in connectivity levels that differ even more from HVs (manuscript under revision). In this study, however, when participants were stratified by degree of antidepressant response it appeared that the MDD participants who responded to ketamine were primarily responsible for the observed lower connectivity compared to HVs in these bands. Combined with the strong evidence of ketamine-induced reductions in connectivity in these bands, this finding may indicate that ketamine induces some compensatory mechanism in responders while disrupting homeostasis in the HVs. These findings are also consistent with the absence of a relationship between MADRS score and changes in beta-band connectivity in an independent cohort of individuals with MDD (28).

In contrast to the α− and β− results, the ketamine-induced changes observed in the θ−θ and θ−β tiles followed a different pattern. At baseline (manuscript under revision), participants with MDD exhibited nominally greater connectivity compared to HVs. The present analysis, however, demonstrated that this result was likely driven by the MDD participants who did not respond to ketamine; these individuals showed significantly greater connectivity in θ− and β− tile pairs regardless of treatment session. In addition, while not significant, it is potentially notable that ketamine responders did not exhibit reductions in connectivity post-ketamine in these bands, and that many exhibited increased connectivity compared to placebo infusion. This may potentially indicate a fundamental alteration in the way the brain biologically responds to NMDA modulation in those who experience an antidepressant effect.

These rather curious results suggest that there may be distinct biological subtypes, based on cortical oscillations, that influence response to ketamine. The ketamine responder subtype is potentially characterized by lower α−α, α−β and β−β connectivity compared to HVs and by resistance to ketamine-induced reductions in δ− and θ− connectivity. In contrast, the ketamine non-responder subtype appears characterized by greater connectivity in all frequency band pairs including δ− or θ− and responds to ketamine with generalized reductions in connectivity. These subtypes may be related to other behavioral domains. For instance, evidence suggests that theta oscillations are involved in control processes (42) and anxiety (43); furthermore, we previously demonstrated that ketamine produces an anxiolytic effect (30). Likewise, alterations in α− and β− connectivity could potentially be related to working and long-term memory processes (44), which ketamine may disrupt (45). These connections, however, are speculative and cannot be established from the data presented herein.

Some studies have attempted to characterize treatment response or non-response in terms of electrophysiological connectivity. For instance, several transcranial magnetic stimulation (TMS) studies using a variety of metrics showed increased theta connectivity in responders (46, 47), no differences at baseline but post-treatment increases in alpha connectivity in responders (48), or more complex patterns across the spectrum (49). An EEG study involving treatment with escitalopram, sertraline, or venlafaxine-XR found reduced resting-state theta connectivity in all MDD participants at baseline, with significantly decreased alpha and nominally decreased theta connectivity post-treatment in male responders only (50). In an electroconvulsive therapy (ECT) study, responders showed reduced resting-state alpha connectivity compared to non-responders (51). While these studies are not consistent with ours, the mechanism of action of each treatment is likely different. These studies are consistent, however, with our suggestion that biological subtypes for MDD in the context of treatment response may involve altered theta-band connectivity.

Our analyses incorporating antidepressant response at either 40 min or Day 1 found a relationship between δ−α, δ−β, and δ−γ connectivity and MADRS score. While ketamine slightly but non-significantly reduced connectivity in these bands overall, HVs with the greatest increase in MADRS score and MDD participants with the greatest decrease in MADRS score both showed slight increases or no change in connectivity. Notably, there were trends toward group differences in mean δ−α and δ−β connectivity (p_uncorr_ = 0.033, p_uncorr_ = 0.021, respectively), with ketamine non-responders showing greater connectivity than HVs regardless of treatment session. Again, these results suggest biological subtypes of MDD conferring responsivity to ketamine treatment. However, these results may also point to subtypes within the HVs as well—those who responded to the ketamine infusion with lowered mood *versus* those that were relatively unaffected.

There is relatively little extant literature on amplitude-amplitude coupling mechanisms between frequencies compared to phase-amplitude coupling, which has been heavily studied, particularly in the theta-gamma range. Recently, however, multiple studies have investigated amplitude correlations between slow (delta or theta) and fast (alpha or beta) oscillations in the context of social behaviors and affective states, particularly anxiety. Most of this research indicates that greater coupling is associated with negative emotional states [reviewed in (52)]. While this is potentially consistent with the observation that MDD non-responders to ketamine exhibited increased δ−α, δ−β, θ−α, and θ−β coupling post-placebo, it is not consistent with our finding that ketamine responders displayed increased δ−α and δ−β coupling. As with our within-frequency findings, however, this may point to distinct biological subgroups within individuals with MDD, potentially related to the presence of significant anxiety. In addition, exploratory *post-hoc* investigations in our HVs revealed trends demonstrating that those who did not show a depressogenic response to ketamine showed greater ketamine-induced decreases in δ−β and δ−γ connectivity compared to those who experienced depressive symptoms (δ−β: t = 2.75, p = 0.014; δ−γ: t = 2.59, p = 0.019), as well as greater connectivity under placebo (δ−α: t = 2.19, p = 0.041; δ−β: t = 1.76, p = 0.093). These results should be interpreted with caution, however, given that the study was not adequately powered for this comparison. Regardless, it is notable that the connectivity changes that correlated with clinical response were primarily cross-frequency, indicating the utility of a multilayer approach.

This study had several limitations. First, because this was part of a complex pharmacologic treatment study in treatment-resistant MDD that was resource-intensive as well as time-intensive for both researchers and participants, the sample size is fairly small. Thus, all findings should be treated with some caution until results can be replicated. Second, the scans took place 6 to 9 h post-infusion rather than during infusion or at the time of peak antidepressant response (generally at Day 1). In addition, by the time of the MEG recording, dysphoric effects in HVs had for the most part resolved. Our choice of timepoint was largely a consequence of scheduling and logistical constraints. However, it could be argued that although Day 1 is the more commonly used timepoint to assess clinical efficacy, the peak of the metabolite concentrations may be closer to 1 to 4 h post-infusion (53). Thus, if metabolites are potentially responsible for MEG response, peak MEG effects may be observable closer to our scan times. While metabolite data were available for these participants, given the breadth of results covered in the present work, we felt that a deeper investigation of metabolite levels on connectivity changes was beyond the scope of the current work. In addition, while many connectivity studies in MDD have incorporated a more comprehensive list of brain regions, our “enriched” set of 34 regions was chosen to reduce the multiple comparisons problem and focus the analysis on the most salient brain regions. Nevertheless, a more exhaustive list of ROIs could be incorporated in future work, although it is not clear that this is warranted.

Some questions are also left unanswered by the current study. First, while the hypothesis that ketamine may exert its effects by enhancing AMPA throughput was reviewed above, this study cannot address that hypothesis directly; however, an ongoing open-label trial (NCT03973268) is using MEG after ketamine infusion with and without pretreatment with the AMPA antagonist perampanel to address this hypothesis directly. Second, while it might be interesting to determine whether any of the ketamine-induced connectivity changes were related to a sustained antidepressant response to ketamine (e.g., > 7 days), we also considered that analysis to be outside the scope of the current manuscript.

In summary, this study extends the current literature on the effects of ketamine in HVs and individuals with MDD. Ketamine generally served to lower connectivity, particularly in the θ−, α−, and β− ranges. There was additional evidence for functional subgroups within the MDD population. Non-responders to ketamine tended to show higher connectivity than controls in the δ– and θ– range, and while they nominally showed reductions in connectivity in response to ketamine, their connectivity values remained above that of HVs. In contrast, MDD participants who experienced an antidepressant response to ketamine tended to show reduced connectivity in the α– and β– range compared to HVs and showed little difference between ketamine and placebo sessions. The identification of depressive subtypes that confer responsiveness to an antidepressant treatment may potentially lead to personalized treatment and faster alleviation of suffering for those with MDD.

## Data Availability Statement

The data that support the findings of this study are available from the corresponding author upon request. The data are not publicly available due to privacy or ethical restrictions.

## Ethics Statement

The studies involving human participants were reviewed and approved by NIH combined CNS IRB. The patients/participants provided their written informed consent to participate in this study.

## Author Contributions

AN: conceptualized and designed the study; conducted the literature review; collected the data; drafted the manuscript; helped interpret the statistical analysis; approved the final version of the manuscript. EB: helped interpret the statistical analysis; edited the manuscript for critical intellectual content; approved the final version of the manuscript. JG: collected the data; assisted in statistical design, analysis, and interpretation; edited the manuscript for critical intellectual content; approved the final version of the manuscript. PT: helped interpret the statistical analysis; edited the manuscript for critical intellectual content; approved the final version of the manuscript. MB: helped interpret the statistical analysis; edited the manuscript for critical intellectual content; approved the final version of the manuscript. CZ: conceptualized and designed the study; provided research supervision; approved the final version of the manuscript.

## Funding

Funding for this work was supported by the Intramural Research Program at the National Institute of Mental Health, National Institutes of Health (IRP-NIMH-NIH; ZICMH002889 and ZIAMH002857).

## Conflict of Interest

CZ is listed as a co-inventor on a patent for the use of ketamine in major depression and suicidal ideation; as a co-inventor on a patent for the use of (2*R*,6*R*)-hydroxynorketamine, (*S*)-dehydronorketamine, and other stereoisomeric dehydro and hydroxylated metabolites of (*R,S*)-ketamine metabolites in the treatment of depression and neuropathic pain; and as a co-inventor on a patent application for the use of (2*R*,6*R*)-hydroxynorketamine and (2*S*,6*S*)-hydroxynorketamine in the treatment of depression, anxiety, anhedonia, suicidal ideation, and post-traumatic stress disorders. He has assigned his patent rights to the U.S. government but will share a percentage of any royalties that may be received by the government.

The remaining authors declare that the research was conducted in the absence of any commercial or financial relationships that could be construed as a potential conflict of interest.
