# Supplementary material for: The Effect of Ketamine on Electrophysiological Connectivity in Major Depressive Disorder
Source: Front Psychiatry. 2020 Jun 10;11:519. doi: 10.3389/fpsyt.2020.00519 (PMC7325927; doi:10.3389/fpsyt.2020.00519)
Supplement: Supplementary file 1 [file Table_1.docx]

*Nugent et al—The Effect of Ketamine on Electrophysiological Connectivity in*

*Major Depressive Disorder*

**Supplementary Figures**

Supplementary Figure S1: Individual tiles for δ-θ and θ−θ connectivity from the full super-adjacency matrix pictured in Figure 4 in the main text. Connections highlighted in color show significant differences in functional connectivity between healthy volunteers (HVs) and participants with major depressive disorder (MDD) who did not experience an antidepressant response to ketamine (MDD-NR). ROIs: regions of interest.


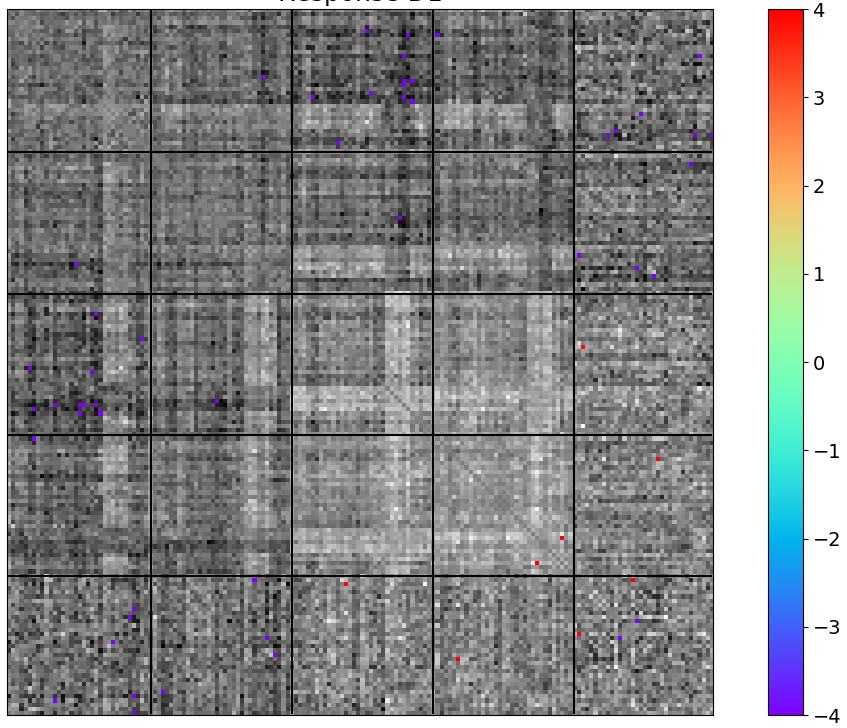


δ

θ

α

β

γ

δ

θ

α

β

γ

Supplementary Figure S2: Connections associated with Montgomery-Asberg Depression Rating

Scale (MADRS) score at Day 1 in participants with major depressive disorder (MDD). The full super-adjacency matrix showing connections that exhibited a significant relationship with percent change in MADRS score at Day 1 in the MDD participants alone.
